# Anti-snake Venom-Induced Kounis Syndrome: A Unique Case in the Emergency Department

**DOI:** 10.7759/cureus.31510

**Published:** 2022-11-14

**Authors:** Aakash Verma, Himanshi Baid, Nakul Sharma, Shuchita Vaya, Sanket M Patel

**Affiliations:** 1 Department of Emergency Medicine, Command Hospital Air Force, Bangalore, Bangalore, IND; 2 Department of Emergency Medicine, All India Institute of Medical Sciences, Rishikesh, Rishikesh, IND; 3 Department of Emergency Medicine, Mahatma Gandhi Medical College and Hospital, Jaipur, Jaipur, IND; 4 Department of Emergency Medicine, Nootan Medical College and Research Centre, Gujarat, IND

**Keywords:** snake bite, allergy and anaphylaxis, emergency department, anti-snake venom, kounis syndrome

## Abstract

Kounis syndrome is the concurrence of acute coronary syndromes associated with allergic or hypersensitivity and anaphylactic or anaphylactoid triggers. Although it is not a rare diagnosis, the different presentations and afflictions of all ages, sex, and racial groups make it a diagnostic challenge. Various triggers include food types, environmental exposure, and drugs. Cases triggered by serum sickness, tetanus antitoxin, and snake bites have been documented in the literature. However, to the best of our knowledge, no case triggered by anti-snake venom (ASV) has been reported yet, as seen in our patient. ASV is composed of refined F(ab)_2 _fragments of immunoglobulin G purified from horse or sheep plasma that has been immunized with the venom of different snake species. Evidence of hypersensitivity has been reported with ASV but not with Kounis syndrome. More so, various other vaccinations have also been associated with Kounis syndrome. We present the case of a 30-year-old male who presented to the emergency department with post-snake bite envenomation and neurological symptoms. After the initiation of the anti-snake venom, the patient’s neurological signs improved. However, the patient developed acute chest pain. His ECG showed transient ST elevation, and cardiac enzymes and serum IgE levels were raised. A diagnosis of Kounis syndrome was made, and the patient was managed accordingly.

## Introduction

Kounis syndrome has been established as the concurrence of acute coronary syndromes with conditions associated with the activation of mast cells, including allergic or hypersensitivity and anaphylactic or anaphylactoid triggers [1]. Kounis syndrome is not as rare a diagnosis as it is ubiquitous, as it affects all age groups, genders, and races. In addition, the myriad of clinical presentations associated with Kounis syndrome makes it a diagnostic challenge, especially in the emergency department [2].

Three variants of Kounis syndrome have been identified: vasospastic allergic angina (type I), allergic myocardial infarction (type II), and stent thrombosis (type III). In the type I variant, patients have normal coronary arteries, which are induced to spasm by an acute allergic reaction as a manifestation of endothelial dysfunction or microvascular angina [2]. A variety of causes that trigger Kounis syndrome have been found, and their number is increasing rapidly. Drugs, several foods, and environmental exposures are the most common triggers [3]. Cases have been described as “serum acute carditis” or “allergic cardiac reactions” due to serum sickness and tetanus antitoxin [3,4]. Cases have also been attributed to snake bites from both the Elapidae and Viperidae species [5,6], however, to the best of our knowledge, there is no case attributing anti-snake venom (ASV) as a specific trigger for Kounis syndrome in the existing literature.

## Case presentation

A 30-year-old male (with no comorbidities) presented to the emergency department with an alleged history of a snake bite on the right foot around six hours after the incident with complaints of pain over the right foot, blurred vision, and difficulty speaking.

The primary assessment revealed a patent airway, a respiratory rate of 20/minute, SpO_2_ of 98% on room air, a pulse rate of 62/minute, and a BP of 126/86 mm Hg. On further examination, there was the presence of ptosis and dysarthria; however, the single breath count was 30. Fang marks were observed at the base of the great toe on the right foot. A provisional diagnosis of snake bite with neurotoxic manifestations was made.

Routine investigations, including blood gas analysis, a 12-lead ECG, a 20-minute whole blood clotting time, a complete blood count, kidney function tests, and a coagulation profile, were done. Two large bore intravenous accesses were obtained, and 10 vials of anti-snake venom (ASV) were diluted in 500 ml of normal saline to be given over 30 minutes after premedication with subcutaneous adrenaline (0.25 mg of a 1:1000 dilution). The first doses of atropine (0.6 mg) and neostigmine (1.5 mg) were also given in view of the neurological symptoms.

**Figure 1:**
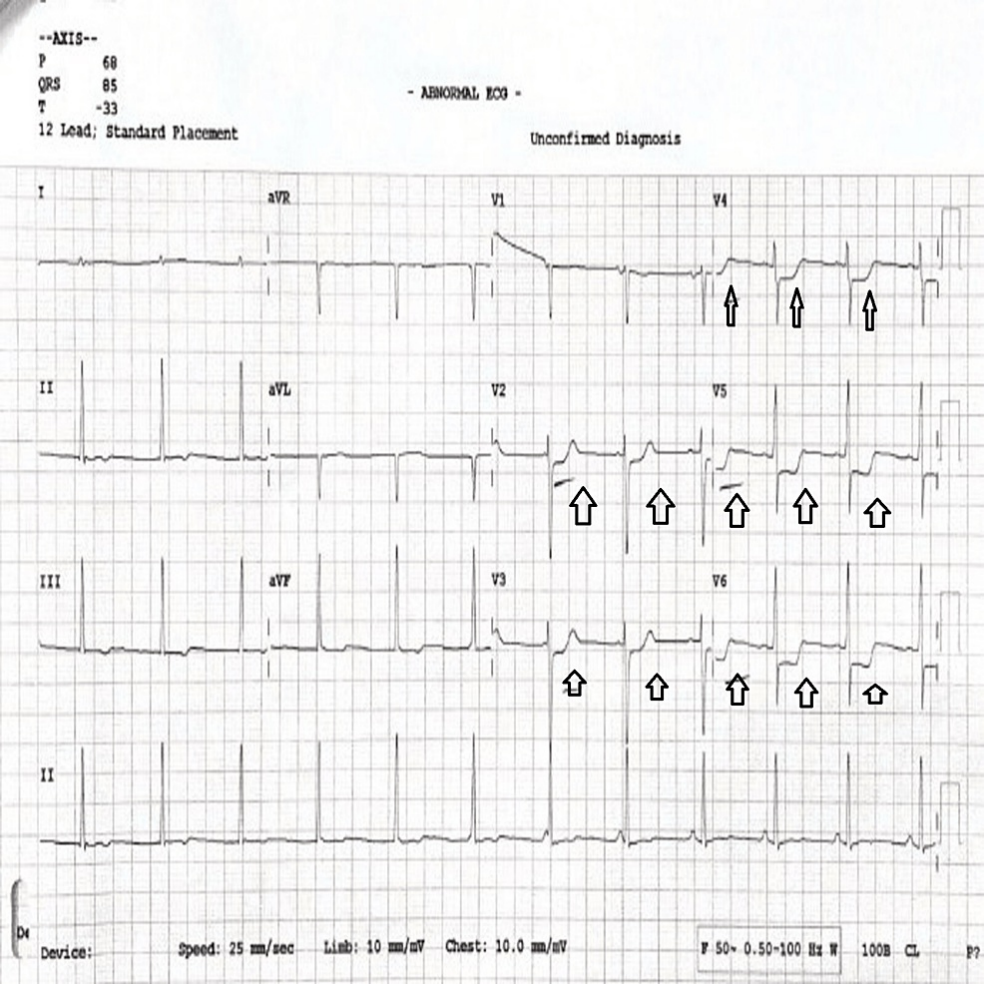
A 12-lead ECG showing ST depression in leads V2-V6.

The patient’s neurological symptoms showed improvement; however, 15 minutes after the completion of ASV, the patient suddenly developed sudden onset retrosternal chest pain, radiating to both arms and associated with sweating. The patient became hypotensive with a BP of 86/50 mm Hg, and his pulse became thready at a rate of 70/minute. About 200 ml of IV crystalloid (normal saline) was given in a bolus over five minutes. A 12-lead ECG was taken immediately (Figure 1).

**Figure 2:**
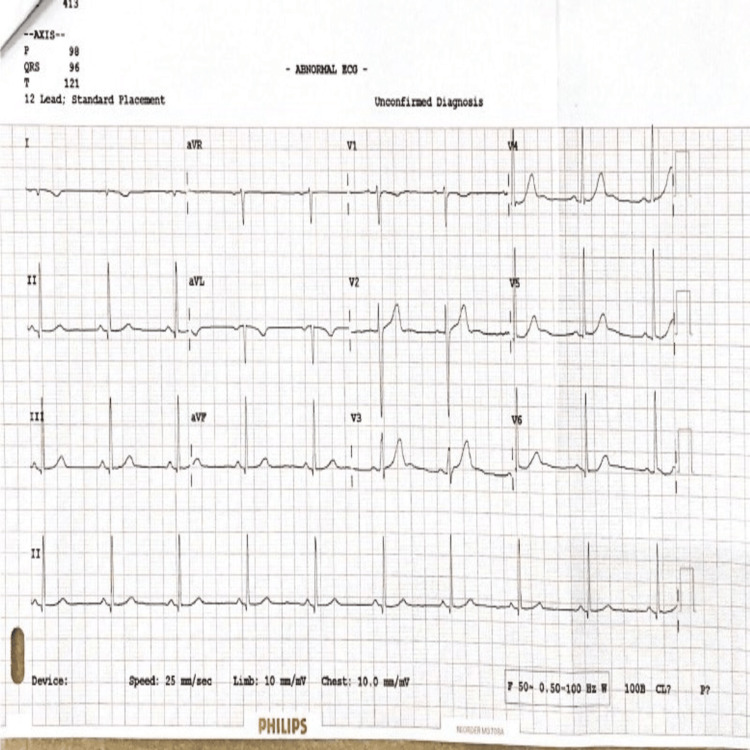
A 12-lead ECG taken at the time of arrival.

The ECG showed new ST segment depression in leads V2-V6 in comparison to the ECG on arrival (Figure 2).

**Figure 3:**
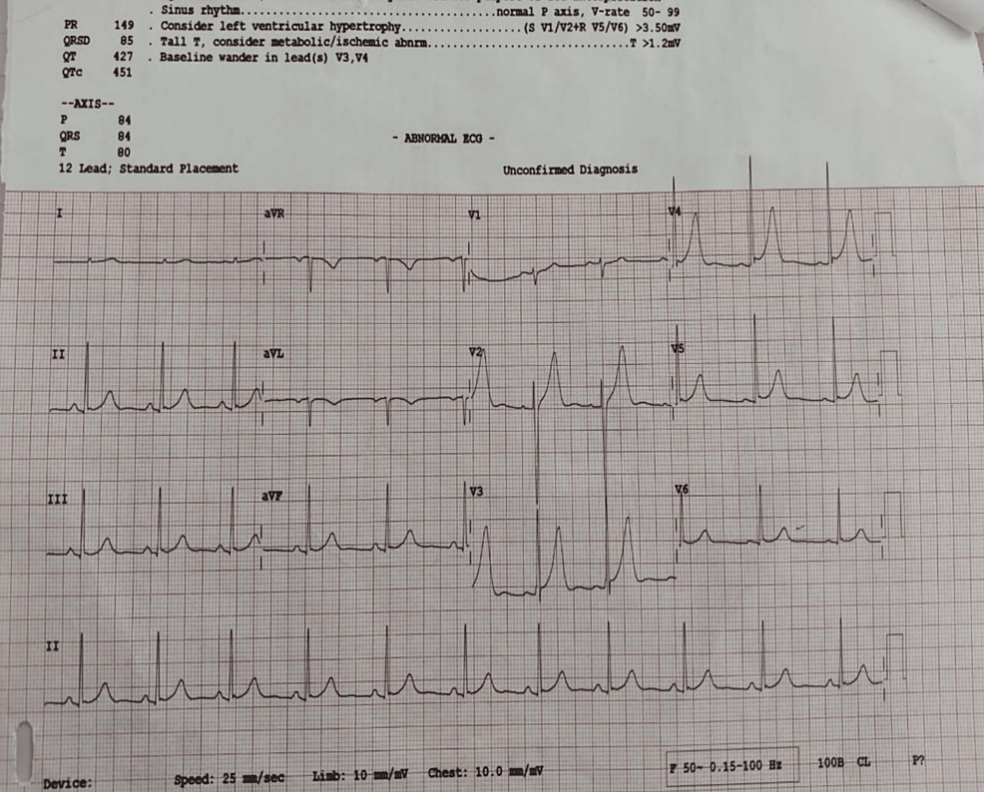
A 12-lead ECG after 12 hours.

We suspected that the administration of ASV triggered an allergic vasospasm of the coronaries; hence, serum IgE levels along with troponin levels were immediately sent, which were both elevated. Point-of-care echocardiography showed septal and lateral wall hypokinesia of the left ventricular wall. The patient was started on vasopressor support with noradrenaline, and the patient was shifted to the intensive care unit after stabilization of blood pressure in lieu with the cardiology team. The patient’s symptoms improved over the next 12 hours, as evidenced by the 12-lead ECG at the time (Figure 3).

The patient was shifted to the ward after 48 hours of ICU stay and discharged from the hospital after a week.

## Discussion

The incidence of Kounis syndrome in the emergency department has been reported to be 19.4 per 100,000 in a year among all admissions in the only prospective study of its kind [7]. The reason for the same is the under-recognition and under-reporting of the same, rather than the rarity of the condition [3]. As emergency physicians, it is of the utmost importance to maintain a high degree of suspicion for the identification of such life-threatening conditions. Another challenge in the emergency department is the treatment of these patients, as there are no specific guidelines for the management of these patients.

The pathophysiology of Kounis syndrome is due to the release of inflammatory mediators during an allergic insult, e.g., histamine, arachidonic acid products, platelet-activating factor, cytokines, and chemokines activate inflammatory cells, including mast cells. The activation and degranulation of mast cells constitute the primary mechanism, producing and releasing inflammatory mediators in the heart tissue and systemic circulation. The mediators can induce coronary vasoconstriction directly, via angiotensin II, or by promoting platelet activation and plaque disruption and the activation of the coagulation cascade, thereby causing vasospasm [2]. Cases of Kounis syndrome due to snake bites (both cobra and viper) have been reported [5,6].

ASV is composed of refined F(ab)_2_ fragments of immunoglobulin G purified from horse or sheep plasma that has been immunized with the venom of different snake species. In India, only polyvalent ASV against the four most important snakes-Indian cobras, Indian common krait, Russell’s viper, and Saw-scaled viper is available. Hypersensitivity reactions are seen in more than 20% of the patients treated with ASV; however, no correlation between ASV and Kounis syndrome has yet been reported [8]. Furthermore, there are several reports of various vaccines leading to Kounis syndrome [2,3].

In our case, our initial dilemma was twofold: first, whether the cardiac ischemia was related to allergic vasospasm, and second, whether the allergic vasospasm was due to the anti-snake venom.

Our patient’s 2D echo showed regional wall motion abnormality corresponding with the ECG findings favoring acute vasospasm rather than direct cardiotoxicity due to the snake bite itself. The highly raised IgE levels supported the presence of an acute allergic reaction. These features, along with the transient ECG changes and resolution of symptoms within 24 hours, would be characteristic of type 1 Kounis syndrome.

## Conclusions

The case highlights a rare complication of Kounis syndrome triggered by the administration of anti-snake venom in a patient with a confirmed neurotoxic snake bite. As primary treating physicians and, in most cases, the first responders, it is imperative for emergency physicians to be aware of such relatively rare albeit immediately manageable complications. Such complications occur more often than reported, thereby, are easily missed. An early and prompt diagnosis of such rare complications would largely benefit the patients by reducing their mortality.
